# Prediction of Minocycline Activity in the Gut From a Pig Preclinical Model Using a Pharmacokinetic -Pharmacodynamic Approach

**DOI:** 10.3389/fmicb.2021.671376

**Published:** 2021-07-09

**Authors:** Quentin Vallé, Béatrice B. Roques, Alain Bousquet-Mélou, David Dahlhaus, Felipe Ramon-Portugal, Véronique Dupouy, Delphine Bibbal, Aude A. Ferran

**Affiliations:** ^1^INTHERES, Université de Toulouse, INRAE, ENVT, Toulouse, France; ^2^Virbac, Carros, France

**Keywords:** antibiotic, intestinal contents, binding, commensal flora, pig model, antimicrobial resistance, microbiota, digestive concentrations

## Abstract

The increase of multidrug-resistant (MDR) bacteria has renewed interest in old antibiotics, such as minocycline, that can be active against various MDR Gram-negative pathogens. The elimination of minocycline by both kidneys and liver makes it suitable for impaired renal function patients. However, the drawback is the possible elimination of a high amount of drug in the intestines, with potential impact on the digestive microbiota during treatment. This study aimed to predict the potential activity of minocycline against *Enterobacterales* in the gut after parenteral administration, by combining *in vivo* and *in vitro* studies. Total minocycline concentrations were determined by UPLC-UV in the plasma and intestinal content of piglets following intravenous administration. In parallel, the *in vitro* activity of minocycline was assessed against two *Escherichia coli* strains in sterilized intestinal contents, and compared to activity in a standard broth. We found that minocycline concentrations were 6–39 times higher in intestinal contents than plasma. Furthermore, minocycline was 5- to 245-fold less active in large intestine content than in a standard broth. Using this PK-PD approach, we propose a preclinical pig model describing the link between systemic and gut exposure to minocycline, and exploring its activity against intestinal *Enterobacterales* by taking into account the impact of intestinal contents.

## Introduction

In the last decades, “old” antibiotics with a narrow spectrum of activity or a low safety margin were left behind in favor of more recent ones. However, the development of antimicrobial resistance led to the emergence of MDR pathogens that dramatically reduced the usefulness of recent antibiotics in the management of community or hospital-acquired infections. At the same time, the decline in the development of new antibiotics limits the therapeutic options available to treat these infections (Wohlleben et al., 2016). In this context, previously neglected antibiotics, some of them still active against bacteria resistant to most recent drugs, need a renewed interest. In recent studies exploring original combinations of old antibiotics for the management of antimicrobial resistance in Gram-negative pathogens, minocycline was identified as a promising companion drug of polymyxin B (Aranzana-Climent et al., 2020; Wistrand-Yuen et al., 2020; Zhao et al., 2020). In this context, the question of the impact of minocycline combination on off-target bacterial populations of the gut microbiota has raised new interest.

Minocycline and other tetracyclines are examples of the rare antibiotics eliminated by both renal and hepatic pathways (Agwuh, 2006), making them suitable for patients with impaired renal function (Welling et al., 1975). However, biliary secretion may lead to high amounts of the drugs in the intestinal tract. For minocycline, about 19–35% of the dose could be found unchanged in feces after administration to patients (Macdonald et al., 1973; Jonas and Cunha, 1982). As a consequence of intestinal elimination, tetracyclines given by oral or parenteral route modify the gut microbiota (as demonstrated *in vitro* in human microbiota and *in vivo* in pigs) (Wagner et al., 2008; Græsbøll et al., 2017; Keerthisinghe et al., 2019; Kromann et al., 2019) and amplify tetracycline-resistant coliform bacteria (Græsbøll et al., 2017; Kromann et al., 2019). Nonetheless, clinical studies of the effects of therapeutic doses on the patients’ microbiota remain relatively scarce (Sullivan et al., 2001; Ley et al., 2006), whereas exploring the link between therapeutic doses and gut exposure to active drug, in terms of time development and magnitude, is essential for understanding the emergence and spread of MDR bacteria inside the gut microbiota.

The drug exposure of the digestive tract depends on the route of administration (oral or parenteral), is driven by plasma exposure through intestinal excretion mechanisms that are specific for each drug (Casals-Pascual et al., 2018), and is influenced by digestive tract anatomy and physiology. Moreover, tetracyclines, like other antibiotics, bind to intestinal contents such as fibers, proteins, and ions (Ahn et al., 2012, 2018), and only the unbound fraction of total intestinal concentrations is available for acting on microorganisms present in the digestive tract. Since the degree of binding depends on the chemical properties of the drug and its affinity for intestinal contents, the percentage of bound drug can greatly vary.

In this context, the present study was designed to describe the gut exposure to minocycline in a pig model, and to explore the relation between gut exposure and antimicrobial effects by taking into account the impact of intestinal contents. For this purpose, we first conducted an *in vivo* PK study in pigs in order to characterize the relationship between blood and gut exposure after intravenous administration of minocycline. Secondly, we explored the impact of local environment on minocycline PD by performing *in vitro* time-kill experiments, with two different *Escherichia coli* strains, in which sterilized intestinal contents (SIC) were used as medium. Finally, we combined these PK and PD components to determine the range of antimicrobial activity against *Enterobacterales* in the gut after minocycline parenteral administration.

## Materials and Methods

### Antimicrobial Drug and Bacterial Strains

Minocycline was used as minocycline hydrochloride purchased at Toronto Research Chemicals Inc., (Toronto, ON, Canada). Minocycline was dissolved in a 5% glucose solution (6.5 mg/mL) for animal experiments and warm deionized water for *in vitro* experiments.

Two *E. coli* strains, the standard ATCC25922 and 2S1F2 isolated from water waste (carrying *CTX-M1*, *sul2*, and *tetA* genes in an IncI1/ST3-CC3 plasmid), were used for *in vitro* experiments. The minimum inhibitory concentration (MIC) of minocycline in mueller hinton broth (MHB) were 0.25 μg/mL for ATCC25922 and 8 μg/mL for 2S1F2.

### Pharmacokinetic Study

#### Animals and Sampling

Nineteen pigs (10 males, nine females) aged 2–4 months and weighing 9.6–13.5 kg at arrival were used for the PK study. They had access to water and feed *ad libitum*.

The experimental protocol was authorized by the French Ministry of Research (Ref: #18953_2019020517093949).

Fifteen pigs received minocycline intravenously in the jugular vein, at the experimental dose (ExD) of 8 mg/kg. For each pig, blood was sampled at slaughter time. Three pigs were sacrificed at each slaughter time (1, 4, 7, 24, or 48 h after administration). They were euthanized by intracardiac administration of pentobarbital (Dolethal^ND^, Vetoquinol, France) following induction of anesthesia with medetomidine (Medetor^ND^, Virbac, France), tiletamine, plus zolazepam (Zoletil^ND^, Virbac, France). Immediately after sacrifice, for each pig, an incision in the intestinal wall was made in order to collect the intestinal content from the small and large intestines. Special attention was paid to avoid blood contamination of samples. Blood samples were centrifuged (3000 × *g*, 10 min, 4°C). Plasma and intestinal contents were stored at −20°C until minocycline quantification. Four control pigs were euthanized (same protocol), and contents from jejunum, ileum, cecum, colon and feces were frozen-stored at −20°C for PD experiments.

#### Minocycline Quantification

Minocycline was quantified in plasma and intestinal contents on a Waters Acquity^TM^ UPLC system (Waters Inc., Milford, MA, United States). Separation was achieved on Acquity^TM^ UPLC CSH Fluorophenyl column (100 × 2.1 mm and 1.7 μm) (Waters Inc., Milford, MA, United States). The liquid chromatographic setting was as follows: mobile phase solvents were H_2_O, 0.1% formic acid (A), and methanol (B). The flow rate was 0.4 mL/min. The gradient program consisted of 10% B (0–4 min) and 10–90% B (4–5 min). The column was heated at 40°C, the photometric diode array detector was set at 350 nm and the injection volume was 25 μL. Retention times for minocycline and tetracycline internal standard (IS) were 1.4 and 1.5 min, respectively.

Plasma samples (100 μL) were mixed with 10 μL of IS (50 μg/mL) and 200 μL 5% trichloroacetic acid. Raw intestinal contents (RIC, 500 mg) were mixed with 500 μL IS and 1 mL 5% trichloroacetic acid (1,400 × g, 2 min, and 10°C). Samples (plasma and RIC) were centrifuged (20,000 × *g*, 10 min, and 4°C) and supernatant (100 μL) was transferred to an autosampler vial and maintained at 4°C for injection. The coefficient of variation (%) of intra-day and inter-day precisions were below 3 and 4%, respectively, and accuracy varied between 97 and 100%. The limit of quantification (LOQ) was 0.05 μg/mL in plasma and the limit of detection (LOD) was 0.25 μg/mL in intestinal contents. More information about the analytical methods can be found in Supplementary Data Sheet 1.

The area under the curve (AUC) of minocycline concentrations in plasma (AUC_plasma_) or digestive contents (AUC_dig24*h*_) over 24 h were calculated by the linear-up/log-down trapezoidal rule.

### Pharmacodynamic Study

#### Killing Curves

RIC from the jejunum, ileum, colon, cecum, and feces of control pigs were diluted in MHB (1 g RIC in 4 mL MHB), filtered, and sterilized to obtain SIC, which were then stored at −20°C until the experiment was initiated. Bacterial suspensions (5 × 10^5^ CFU/mL) of *E. coli* strains were exposed to different ranges of minocycline concentrations, from 0.0625 to 1,024 μg/mL. Each suspension was sampled after 0, 1, 2, 4, 7, and 24 h of incubation at 37°C to count viable bacteria. The samples were centrifuged (3,000 × *g*, 3 min, and 4°C) and the pellets were suspended in the same volume of saline. Bacteria were counted in triplicate on tryptic soy agar supplemented with magnesium heptahydrate sulfate and activated charcoal after overnight incubation at 37°C. The LOQ was set to 33 CFU/mL.

#### Modeling of Mean Inoculum Growth

From each time-kill study, AUC of bacterial counts over time were calculated by the trapezoidal rule and divided by 24 h, giving a mean inoculum size (I*x*) for each *x* minocycline concentration. The mean inoculum growth ΔI(*x*) over 24 h was defined as ΔI(*x*) = I_*x*_–I_BASAL_, where I_BASAL_ is the initial inoculum size.

Mean inoculum growth was modeled as a function of minocycline concentration, using a previously published re-parametrized sigmoid E_max_ model (Regoes et al., 2004; Ferran et al., 2013; Lhermie et al., 2015):

(1) $$△\,I\,\left(x\right)=△\,I_{M\,A\,X}-\frac{\left(△\,I_{M\,A\,X}-△\,I_{M\,I\,N}\right)\times {\left(x/E\,C_{-3\,l\,o\,g}\right)}^{y}}{\left(\left(-3-△\,I_{M\,I\,N}\right)/\left(△\,I_{M\,A\,X}+3\right)\right)+{\left(x/E\,C_{-3\,l\,o\,g}\right)}^{y}}$$

where, ΔI(*x*) (Log10 CFU/mL) is the mean inoculum growth for a minocycline concentration *x*, ΔI_MAX_ and ΔI_MIN_ are the maximal and minimal inoculum growth, obtained without antibiotic (ΔI_MAX_) or after exposure to the highest minocycline concentration (ΔI_MIN_), *x* (μg/mL) is the minocycline concentration, EC_–3log_ (μg/mL) is the minocycline concentration associated to a 3 Log10 (99.9%) reduction of I_BASAL_ (ΔI(EC_–3*log*_) = −3), and γ is the sigmoid coefficient of the curve. EC_–3log_ was identified as the lowest bactericidal concentration in the rest of the article.

### Prediction of Minocycline Activity in the Gut

The pharmacodynamic model described by Equation 1 was used to predict minocycline activity in each digestive segment for concentrations in gut segments corresponding to selected doses.

The mean minocycline concentrations (μg/mL) over 24 h in each gut segment after administration of the experimental dose (ExD) was calculated using Equation 2:

(2) $$C_{d\,i\,g\,E\,x\,D}=\frac{A\,U\,C_{d\,i\,g\,24\,h}}{24}$$

where, AUC_dig 24h_ (μg.mL.h^–1^) is the AUC of total minocycline concentrations in the pig digestive contents over 24 h after administration of the ExD.

The exposure of pig gut segment to minocycline after administration of a dose equivalent (in terms of plasma exposure) to a human dose (HuD) was calculated using Equation 3:

(3) $$C_{d\,i\,g\,H\,u\,D}=\frac{A\,U\,C_{p\,l\,a\,s\,m\,a\,H\,u\,D}}{A\,U\,C_{p\,l\,a\,s\,m\,a\,E\,x\,D}}\times C_{d\,i\,g\,E\,x\,D}$$

where, AUC_plasmaExD_ and AUC_plasmaHuD_ (μg.mL.h^–1^) are the AUC (24 h) of minocycline plasma concentrations in pigs receiving the ExD, and the AUC (24 h) of minocycline plasma concentrations in human patients receiving an intravenous dose of minocycline (HuD), respectively. According to the current recommendations (Melinta Therapeutics, 2020), the HuDs selected for predictions were 200 and 400 mg and the corresponding AUC_plasmaHuD_ was obtained from Lodise et al. (2020).

## Results

### Pharmacokinetic Study

#### Minocycline Quantification

**FIGURE 1:**
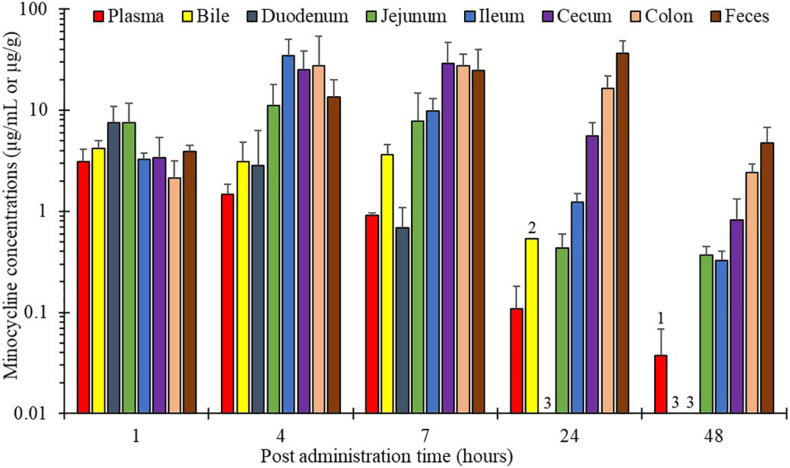
Minocycline concentrations (mean ± SD) over time in plasma and in different intestinal matrices of piglets after a single intravenous administration of 8 mg/kg minocycline (*n* = 3). (1) One datum below the LOQ. (2) Two data below the LOQ. (3) Three data below the LOQ. LOQ_plasma_ = 0.05 μg/mL, LOD_intestinal matrices_ = 0.25 μg/mL or μg/g.

Minocycline concentrations in plasma and the different gut segments (bile, duodenum, jejunum, ileum, cecum, colon, and feces) after intravenous administration of 8 mg/kg minocycline to pigs are shown in Figure 1. From 4 h after administration, minocycline concentrations increased in gut segments (except duodenum), and not in plasma. Minocycline concentrations remained high over the experimental period in cecum, colon and feces, with concentrations over 0.8 ± 0.5 μg/mL at 48 h, whereas concentrations in plasma, bile, and duodenum were below the LOQ at this same time. The maximal digestive concentrations were 1.4- to 10-fold higher than the maximal concentration obtained in plasma and measured between 1 and 24 h, depending on the gut segment (Table 1).

**TABLE 1:** Maximal concentration (C_max_, mean ± SD of three measures) and time at which C_max_ was observed (T_max_) in plasma and gut segments after a single intravenous administration of minocycline (8 mg/kg BW) to 15 pigs.

	**Plasma**	**Bile**	**Duodenum**	**Jejunum**	**Ileum**	**Cecum**	**Colon**	**Feces**
Cmax (μg/mL or μg/g)	3.0 ± 1.0	4.2 ± 0.2	7.6 ± 3.4	11.1 ± 6.9	34.6 ± 15.6	29.0 ± 17.7	27.6 ± 8.4	36.5 ± 12.0
Tmax (hours)	1	1	1	4	4	7	7	24

From jejunum to rectum (feces), the AUC_dig_/AUC_plasma_ ratio increased, ranging between 6 and 39, implying that average minocycline concentrations over 24 h were, on average, 6- to 39-fold higher in digestive segments than in plasma (Table 2).

**TABLE 2:** AUC_plasma_, AUC_dig_, and AUC_dig_/AUC_plasma_ ratio over 24 h following a single intravenous administration of 8 mg/kg minocycline to 15 pigs.

	**AUC_plasma_ (μg.h/ml) or AUC_dig_ (μg.h/g)**	**AUC_dig_/AUC_plasma_**
Plasma	15.2	–
Bile	44.6	2.9
Duodenum	18.0	1.2
Jejunum	86.1	5.7
Ileum	146	9.6
Cecum	325	21.4
Colon	466	30.7
Feces	588	38.7

### Pharmacodynamic Studies

#### Analysis of Minocycline Activity in SICs

**FIGURE 2:**
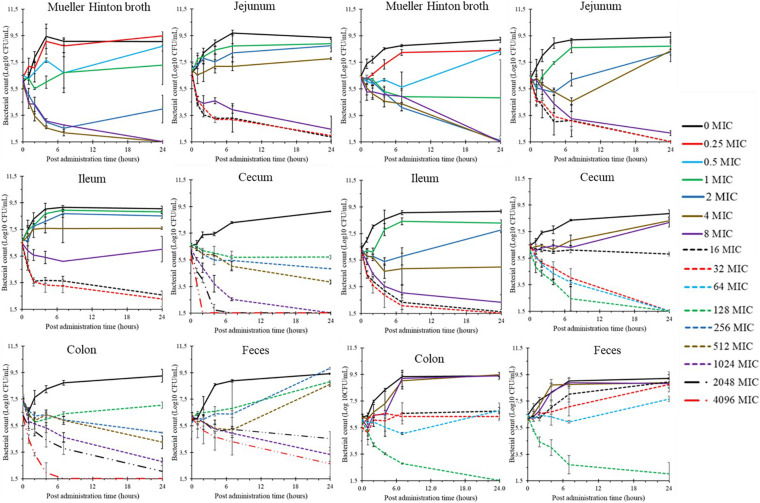
Time-kill curves of minocycline against ATCC25922 (left) and 2S1F2 (right) *Escherichia coli* strains tested in MHB and SIC (from jejunum, ileum, cecum, colon, and feces); points are mean ± SD (*n* = 3 for each matrix and concentration).

**FIGURE 3:**
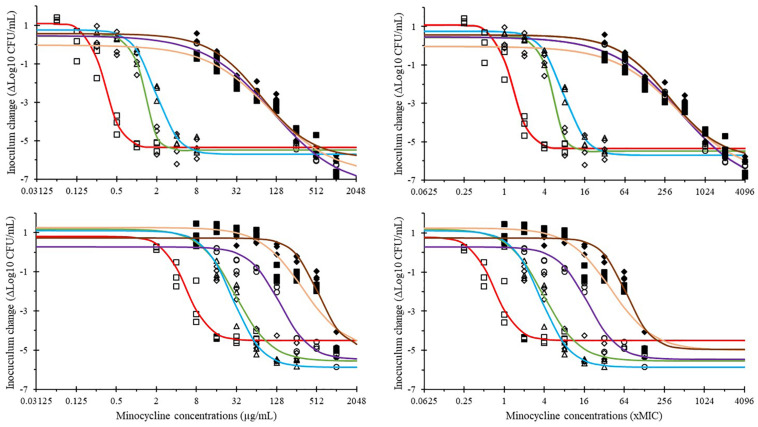
Modeling of the mean inoculum change (ΔLog10 CFU/mL) in function of minocycline concentrations expressed in μg/mL (left) and MIC fold (right) in different media for two *E. coli* strains ATCC25922 (top) and 2S1F2 (bottom); media were MHB (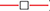), SIC jejunum (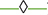), SIC ileum (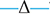), SIC cecum (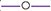), SIC colon (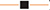), and SIC feces (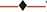).

The time-kill curves of minocycline against ATCC25922 and 2S1F2 strains in MHB and the different SICs are presented in Figure 2. The relationships between mean inoculum changes and minocycline concentrations are presented in Figure 3. The data were fitted with the pharmacodynamic model **(Equation 1)** and the parameters are presented in Tables 3, 4. In all media (MHB and SICs), minocycline exhibited bactericidal activity against both strains even if concentrations required for bactericidal effect were much higher in the SICs (Figure 3).

**TABLE 3:** Mean ± SD pharmacodynamic parameters calculated with Equation 1 (sigmoid E_max_ model), describing killing effects of minocycline on ATCC25922 in different matrices (mueller hinton broth (MHB) and sterilized intestinal contents (SIC), from jejunum, ileum, cecum, colon, and feces).

**Medium**		**Basal inoculum size (Log10 CFU/mL)**	**EC_–3Log_**			**ΔImax**	**ΔImin**
				
			**μg/mL**	**xMIC**	**γ**	**(Log10 CFU/mL)**	
MHB		6.2 ± 0.1	0.4 ± 0.1	1.6 ± 0.2	3.3 ± 0.9	1.1 ± 0.4	−5.4 ± 0.3
	Jejunum	6.8 ± 0.4	1.4 ± 0.2	5.5 ± 0.6	4.7 ± 1.1	0.4 ± 0.5	−5.5 ± 0.7
	Ileum	6.6 ± 0.1	2.2 ± 0.2	8.7 ± 0.2	2.5 ± 0.6	0.7 ± 0.1	*−*5.7 ± 0.7
SIC	Cecum	6.4 ± 0.1	81.7 ± 2.8	326.6 ± 11.2	0.9 ± 0.1	0.5 ± 0.1	−7.5 ± 0.2
	Colon	7.1 ± 0.1	91.3 ± 6.0	365.2 ± 23.9	0.6 ± 0.1	0.1 ± 0.1	−11.2 ± 1.8
	Feces	6.1 ± 0.1	87.5 ± 11.7	350.1 ± 46.9	1.1 ± 0.1	0.6 ± 0.2	NA*

EC_–__3 log_, lowest antibiotic concentration reducing basal bacterial population by 3 Log10 CFU/mL; γ sigmoid coefficient of curve; ΔImax, maximum growth compared with basal inoculum; ΔImin, minimum growth (maximum killing) compared with basal inoculum; MIC, minimum inhibitory concentrations (MHB); *, not assessable.

**TABLE 4:** Mean ± SD pharmacodynamic parameters calculated with Equation 1 (sigmoid E_max_ model) describing killing effects of minocycline on 2S1F2 in different matrices (MHB and SIC, from jejunum, ileum, cecum, colon, and feces).

**Medium**		**Basal inoculum size**	**EC_–3Log_**			**ΔImax**	**ΔImin**
				
		**(Log10 CFU/mL)**	**μg/mL**	**xMIC**	**γ**	**(Log10 CFU/mL)**	
MHB		6.3 ± 0.1	8.1 ± 2.7	1.0 ± 0.3	1.0 ± 0.3	2.5 ± 0.3	0.8 ± 0.1
	Jejunum	6.2 ± 0.2	41.8 ± 11.3	5.2 ± 1.4	1.6 ± 0.2	1.2 ± 0.2	−5.6 ± 0.4
	Ileum	6.2 ± 0.2	34.0 ± 7.2	4.3 ± 0.9	2.0 ± 0.1	1.1 ± 0.3	−5.9 ± 0.2
SIC	Cecum	6.3 ± 0.1	166.0 ± 14.0	20.7 ± 1.7	1.9 ± 0.2	0.3 ± 0.1	−5.5 ± 0.4
	Colon	6.1 ± 0.1	559.5 ± 20.0	69.9 ± 2.5	1.4 ± 0.2	1.3 ± 0.3	NA*
	Feces	6.3 ± 0.2	721.0 ± 86.1	90.1 ± 10.8	2.3 ± 0.3	0.7 ± 0.2	NA*

EC_–__3 log_, lowest antibiotic concentration reducing basal bacterial population by 3 Log10 CFU/mL; γ sigmoid coefficient of curve; ΔImax, maximum growth compared with basal inoculum; ΔImin, minimum growth (maximum killing) compared with basal inoculum; MIC, minimum inhibitory concentrations (MHB); *, not assessable.

In MHB, the minocycline EC_–3log_ were 0.4 and 8.1 μg/mL for ATCC25922 and 2S1F2 strains, respectively, which corresponds to 1.6- and 1.0-fold their respective MIC (Tables 3, 4). Because of the shift to the right of the concentration-effect curves in SICs compared to MHB, these concentration values were associated with net inoculum growth in all SICs.

The decrease of minocycline activity was of similar magnitude in jejunum and ileum SICs for both bacterial strains (Figure 3), with EC_–3log_ varying from 5.5- to 8.7-fold the MIC for ATCC25922, and from 5.2- to 4.3-fold the MIC for 2S1F2. Conversely, the situation was contrasted between the two strains in cecal, colonic and fecal SICs. Indeed, whereas, for ATCC25922, the concentration-effect curves were very close (Figure 3, upper panels) with EC_–3log_ ranging from 326- to 365-fold the MIC (Table 3), and curves were shifted for 2S1F2 between the three SICs (Figure 3, lower panels) with EC_–3log_ ranging from 21- to 90-fold the MIC (Table 4).

#### Prediction of Minocycline Activity in Gut Segments for Human Equivalent Doses

The PD model was used to predict the activity of minocycline concentrations in pig gut segments, corresponding to the HuDs of 200 and 400 mg. The results are presented in Table 5.

**TABLE 5:** Predicted activity (ΔLog10 CFU/mL) against ATCC25922 and 2S1F2 *Escherichia coli* strains of the mean minocycline concentrations in pig gut segments corresponding to HuDs of 200 and 400 mg.

		**Small intestine**		**Large intestine**		
**Dose (mg)**	**Strain**	**Jejunum**	**Ileum**	**Cecum**	**Colon**	**Rectum (Feces)**
HuD = 200	ATCC25922	−5.6	−5.6	−1.1	−1.8	−1.6
	2S1F2	0.8	0.4	0.1	1.0	0.7
HuD = 400	ATCC25922	−5.5	−5.7	−2.0	−2.7	−2.8
	2S1F2	0.1	−1.1	−0.3	0.6	0.6

*HuD, human doses.*

When considering gut segments of the large intestine (cecum, colon, and rectum), minocycline concentrations corresponding to HuD = 200 mg were associated with slight inoculum reductions (−1.1 to −1.8 Log10) for the more susceptible strain (ATCC25922), and bacteriostatic or slight net growth effect (0.1–1.0 Log10) on the less susceptible strain (2S1F2). For the HuD = 400 mg, the inhibiting effect on ATCC25922 was more pronounced (−2.0 to −2.8 Log10), whereas nearly bacteriostatic or slight net growth (−0.3 to 0.7 Log10) were still observed on 2S1F2.

## Discussion

The objective of this study was to determine pig digestive tract exposure to minocycline and explore its associated activity against intestinal *E. coli*. We developed a model that combined *in vivo* PK and *in vitro* PD data, and in linking plasma and intestinal minocycline concentrations, allowed prediction of the range of minocycline activity against *Enterobacterales* in the gut for any parenteral dose.

The extent and duration of pig exposure to minocycline were assessed after intravenous administration by measuring total minocycline concentrations in plasma and in different gut segments. After parenteral administration, the gut transfer of any drug is driven by plasma concentrations, which undergo a series of active/passive mechanisms of secretion and/or re-absorption that are drug-specific (Casals-Pascual et al., 2018). Once the digestive tract is reached, the extent and duration of gut exposure is the result of a concentrating process into intestinal fluids, and of gut motility that slows drug progression into the distal segments. Consequently, total minocycline concentrations in plasma and gut segments exhibited divergent time developments (Table 1), with concentrations in gut segments (except duodenum, biliary secretion occurring downstream) increasing to reach maximal levels within 24 h, whereas plasma concentrations continuously decreased. Finally, the overall total minocycline concentrations in the gut segments (except duodenum) were 6- to 39-fold higher than in plasma (Table 2).

In parallel to PK, we explored minocycline PD at the intestinal level with *in vitro* time-kill studies. We used two *E. coli* strains, minocycline MICs belonging to wild type (ATCC25922) and non-wild type (2S1F2) *Enterobacterales* populations (EUCAST, 2009). The time-kill experiments were performed in MHB and intestinal contents, diluted, and sterilized (SICs). The objective of performing time-kill experiments in SICs was to assess the influence of the local environment of intestinal contents on the antibacterial activity of minocycline.

As MHB was characterized by the absence of drug binding to its constituents, the activities measured in this medium are representative of the unbound drug (Beer et al., 2009). In the gut, minocycline (like other drugs) can bind to different components of intestinal contents, and because composition of intestinal contents varies, the proportion of bound drug can greatly vary, depending on location in the digestive tract (Ahn et al., 2012, 2018). Consequently, we prepared SICs from different gut segments: jejunum and ileum, which are part of the small intestine, cecum and colon, which belong to the large intestine, and the rectum, containing feces. The sterilization process, which aimed at eliminating resident microbiota while preserving the chemical constituents, was supposed to have a minor impact on the binding capacity of intestinal contents, as previously shown in steam-sterilized and non-sterilized feces (Ahn et al., 2012, 2018).

The analysis of mean inoculum changes in MHB (Figure 3 and Tables 3, 4) showed very similar antibacterial activity of unbound minocycline concentrations against both *E. coli* strains, when standardized by their respective MICs.

Minocycline activity in SICs from the small intestine (jejunum and ileum) was decreased compared to MHB, as illustrated by the shift to the right of the mean inoculum changes vs. minocycline concentration curves (Figure 3). The EC_–3log_ of minocycline against both *E. coli* strains ranged within 4.3–8.7 times the MICs in jejunal and ileal SICs (Tables 3, 4). A similar shift between MHB and jejunal/ileal SICs was previously observed with another antibiotic (Ferran et al., 2013).

When considering minocycline activity in SICs from large intestine segments (cecum and colon) and feces, the analysis of mean inoculum changes vs. minocycline concentration curves revealed two interesting features (Figure 3). One finding included a shift to the right of the curves, illustrating additionally decreased minocycline activity compared to jejunal/ileal SICs. In contrast with these media (and MHB), the mean inoculum changes curves in cecal, colonic and fecal SICs showed different shapes between the two *E. coli* strains. More precisely, the three curves were very close for ATCC25922, with EC_–3log_ ranging within 326–365 times the MIC (Figure 3 and Table 3), whereas the curves for 2S1F2 were clearly separated with EC_–3log_ ranging within 21–90 times the MIC (Figure 3 and Table 4). Such contrast between the two *E. coli* strains reveals the complexity of the interactions between intestinal contents, antibiotic, and bacteria.

One factor explaining the decreased activity of minocycline in SICs compared to MHB is its binding to the components of the SICs, resulting in a decrease of unbound concentrations of minocycline. Ahn et al. (2018) assessed the binding of tetracycline in 25% (w/v) diluted human sterilized feces. They estimated an unbound fraction of approximately 40%. In our study, under the double assumption that minocycline activity in SICs is due to unbound concentrations and that these concentrations have the same effect as total concentrations in MHB, the ratio of EC_–3log_ obtained in MHB and fecal SIC should result in the unbound fraction of minocycline in feces. The values of this ratio are 0.4–1.1% for the two *E. coli* strains, which is very small compared to a 40% unbound fraction. These discrepancies suggest that drug binding is not the sole factor influencing minocycline activity in SICs. Moreover, the contrasted features of minocycline activity against the two *E. coli* strains in cecal/colonic/fecal SICs reinforce this hypothesis, because the sole binding factor cannot explain that EC_–3log_ were almost identical with one strain, and not with the other one.

It should be noticed that minocycline has been shown to exhibit non-linear binding to plasma protein (Zhou et al., 2017b; Dorn et al., 2018). However, the drug binding in intestinal contents, and particularly in the distal gut segments (large intestine), occurs in an environment very different to that of plasma: (1) it does not involve the same proteins (if any), but rather constituents of the matrix such as cellulose, and (2) it occurs in less hydrated environment, and with different characteristics of molecular interaction (adsorption). Therefore, extrapolating plasma binding characteristics to large intestine conditions should be considered with caution.

We suggest that an interaction between bacteria and intestinal contents that influences the antibacterial action of minocycline also exists. Even if we did not explore the mechanisms of this interaction, the attachment of bacteria to some constituents of the matrix of intestinal contents could potentially modify their phenotypes and decrease their susceptibility to antibiotics, as has been established with biofilms (de Vos, 2015; Tytgat et al., 2019). The combination of factors decreasing concentrations available for microorganisms and factors reducing bacteria susceptibility to unbound antibiotic could be responsible for the effects observed in cecal/colonic/fecal SICs.

The model developed in pigs can be used to predict, for a parenteral dose used in humans, the range of minocycline activity in the gut, and particularly on *E. coli*. The first step of the prediction consists of determining local minocycline concentrations, by interspecies scaling using clearance ratio to determine in pigs the plasma exposure (AUC) corresponding to the HuD, followed by the scaling of plasma to gut exposure. To be valid, the approach requires the assumption of dose-proportionality of minocycline plasma PK, as well as proportionality between plasma and digestive tract exposures in both pigs and humans. Although no data are available for pigs to our knowledge, published data indicate a dose proportionality for minocycline in human’s and in mice (Mitchell and Plott, 2012; Zhou et al., 2017a). The activity of minocycline concentrations in the gut was then predicted using the PD model developed for each gut segment. The predicted minocycline activities showed different presentations between the two *E. coli* strains. Indeed, the most susceptible strain would be extensively killed in jejunum/ileum, while the killing activity of minocycline would be limited in the cecum/colon/feces, as well as for the less susceptible strain along the digestive tract.

Regarding the limits of our model, it can be stressed that *in vitro* time-kill studies were performed on a homogeneous bacterial population at an initial density of 10^5^ CFU/mL. The situation is more complex *in vivo*, as the microbiota are variable along the intestinal tract, in terms of density and diversity: 10^3^–10^4^ bacteria/mL and lower diversity in the upper segments, and 10^10^–10^11^ bacteria/mL and higher diversity in the large intestine (Hillman et al., 2017). Therefore, predicted minocycline activities in the latter gut segments are probably overestimated, as antibacterial activity of antibiotics is decreased for high bacterial populations (10^8^ CFU/mL) (Ferran et al., 2009; Kesteman et al., 2009; Vasseur et al., 2014). Moreover, recent works indicated that antibiotic activity can be modified when target bacteria are embedded in a natural bacterial community, as is the case of gut microbiota (Kraupner et al., 2018; Klümper et al., 2019).

Another limitation of our investigation is that we evaluated minocycline activity on a limited part of gut microbiota (*E. coli* stains, as representatives of *Enterobacterales*) and by measuring only bacterial killing, whereas antibiotics can affect bacteria in many ways. In particular, sub-bactericidal or sub-inhibitory concentrations of antibiotics can impact the physiology of living bacteria, as in the horizontal transfer of conjugative or integrative genetic elements (Doucet-Populaire et al., 1991; Bahl et al., 2004). We will further investigate the impact of minocycline on the gut microbiota through Whole Genome Sequencing.

Nevertheless, the model proposed in this study is a preliminary attempt to integrate a part of the complexity of the mechanisms that relate antibiotic plasma exposure to effects on gut microbiota, utilizing a PK/PD approach. Further improvements of this PK/PD approach should focus on finer modeling of the time development of the PK and PD processes, by integrating the antibiotic transit times along the gut on the PK side, as well as bacterial growth models for PD modeling of time-kill curves.

In conclusion, we developed a PK/PD approach in a preclinical pig model to characterize the link between plasma and intestinal minocycline concentrations, and to explore its associated activity against intestinal *Enterobacterales* by taking into account the influence of intestinal contents. Such a model should become a promising tool for exploring the off-target effects on the gut microbiota of any antibiotic dosage, either during the re-evaluation process of old antibiotics or during the preclinical development of new drugs.

## Data Availability Statement

The raw data supporting the conclusions of this article will be made available by the authors, without undue reservation.

## Ethics Statement

The animal study was reviewed and approved by Comité d’Éthique de Pharmacologie-Toxicologie de Toulouse Midi-Pyrénées (CEEA N°86, Toxcométhique).

## Author Contributions

QV, BR, AB-M, DD, FR-P, VD, DB, and AF: conceptualization, methodology, writing—review, and editing. QV and DD: data collection. QV, AB-M, and AF: data curation and validation. QV, AB-M, DB, and AF: formal analysis. AB-M: funding acquisition. QV and AF: writing—original draft. All authors contributed to the article and approved the submitted version.

## Conflict of Interest

QV is a Virbac employee, however, Virbac was not involved in the experiment design, data analysis or manuscript writing. The remaining authors declare that the research was conducted in the absence of any commercial or financial relationships that could be construed as a potential conflict of interest.
