# Supplementary material for: Prediction of Minocycline Activity in the Gut From a Pig Preclinical Model Using a Pharmacokinetic -Pharmacodynamic Approach
Source: Front Microbiol. 2021 Jul 9;12:671376. doi: 10.3389/fmicb.2021.671376 (PMC8299485; doi:10.3389/fmicb.2021.671376)
Supplement: Supplementary file 1 [file Data_Sheet_1.PDF]

<u>Appendix</u>: Validation results and chromatograms of minocycline in pig plasma and intestinal contents

The LC/UV method for quantification of minocycline was validated in pig plasma according to the European Medicines Agency guidelines (EMEA/CHMP/EWP, 2011) in terms of calibration curve, limit of quantification, intra and inter-day repeatabilities and dilution. Selectivity was evaluated by comparing six blank plasma chromatograms with chromatograms at the lower limits of quantification (LOQ) (figure 1). No interference were found at the time of retention of minocycline.

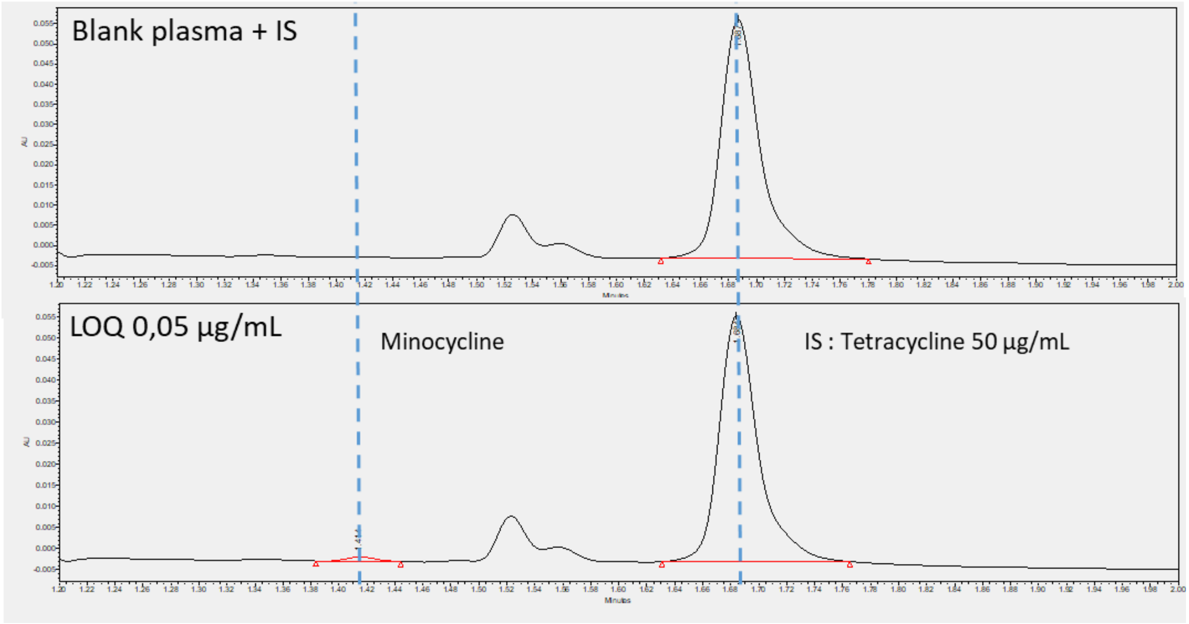

Figure 1: Chromatograms of extracted pig blank plasma spiked with internal standard (IS : 10 µL of tetracycline at 50 µg/mL) and extracted pig plasma spiked at the LOQ (0.05 µg/mL) with IS.

The LLOQ was evaluated with six replicates of plasma samples spiked at 0.05 µg/mL and was set as the lowest concentration level of the calibration curve that could be quantified with acceptable precision and accuracy, lower than 20% and within 80-120%, respectively. Seven calibration points at concentr ations ranging from 0.05 to 10 µg/mL were extracted in triplicates the first day. Both linear ($Y = aX + b$) and quadratic ($Y = aX^2 + bX + c$) models were tested with 1, 1/X and $1/X^2$ (X = nominal concentration) weightings with these resulting calibration curves. Three approaches were tested to select the best calibration model: 1) inspection of the residual distribution plot against nominal concentrations, 2) a lack-of-fit test to check the goodness-of-fit of the model and 3) calculation of the relative concentration residuals (RCR%) between the nominal concentration and the concentration obtained with the model, which

should be lower than ± 15%. Intra-day and inter-day precisions and accuracies were calculated on three different days with six replicates of QC samples at three concentration levels (Low= 0.15 µg/mL, Mid 1.5 µg/mL and High= 8 µg/mL) covering the range of the calibration curve concentrations (0.05 – 10 µg/mL). Intra and inter-day precisions were expressed with a coefficient of variation percent (CV%) and calculated by ANOVA. The fivefold dilution was checked with three QC at 40 µg/mL to allow sample quantification above 10 µg/mL. The stability of extracted minocycline in the autosampler was assessed by reanalyzing the QC samples stored at 15°C and in darkness at t0, 4, 8, 12 and 24H. All these results were reported in table 1.

Table 1: Validation results of minocycline in pig plasma

Nominal concentration (µg/mL)		Mean (µg/mL)	Mean accuracy %	Accuracy range %	Precision (CV%)	
					Within-day	Between-day
Calibration curve (n= 5)[a]						
	0.05	0.053	106%	99% - 116%	9%	7%
	0.1	0.096	96%	93% - 101%	5%	3%
Quadratic	0.5	0.495	99%	96% - 101%	2%	2%
model,	1	0.995	99%	93% - 105%	3%	4%
weight 1/X	2	1.956	98%	93% - 103%	5%	4%
	5	5.087	102%	99% - 106%	2%	3%
	10	9.967	100%	98% - 101%	1%	1%
LOQ (n = 6)						
	0.05	0.052	104%	101% - 109%	3%	--
QC (n = 6 x 3)						
Low	0.15	0.149	100%	94% - 106%	3%	4%
Mid	1.5	1.497	100%	94% - 105%	2%	4%
High	8	7.726	97%	93% - 100%	2%	2%
Autosampler stability[b]						
Low QC (n=3)						
4H	0.15	0.156	104%	--	6%	--
8H	0.15	0.158	105%	--	0.2%	--
12H	0.15	0.158	106%	--	1%	--
24H	0.15	0.161	108%	--	3%	--
Mid QC (n=3)						
4H	1.5	1.533	102%	--	3%	--
8H	1.5	1.497	100%	--	4%	--
12H	1.5	1.563	104%	--	4%	--
24H	1.5	1.610	107%	--	2%	--
High QC (n=3)						
4H	8	7.773	97%	--	0.2%	--
8H	8	7.911	99%	--	0.4%	--

12H	8	7.972	100%	--	1%	--
24H	8	8.137	102%	--	0.3%	--
Dilution (n = 3)						
dilution 1/5	40	38.917	97%	--	4%	--

[a]Calibration model and intra-day precision were determined with 3 replicates for the first day and inter-day precision was evaluated on each concentration for 3 consecutive days.

[b]Autosampler stability was evaluated in the darkness and at 15 °C

For intestinal contents, the calibration curve was prepared in aqueous solution from 0.25 µg/mL to 100 µg/mL. Two replicates per matrix of QC at 10 µg/mL were extracted over two different days and assayed on the daily calibration curve with a linear model weighted by $1/X^2$ (X = concentration). The recovery of each matrix was calculated by comparing the concentration of QC sample extracted from the matrix and calculated from the aqueous calibration curve with its nominal concentration, recovery of each matrices were reported in table 2.

Table 2: Minocycline recoveries in the intestinal contents.

Intestinal Contents (n=2)	Nominal Concentration (µg/g)	Minocycline Day 1 (µg/g)	Minocycline Day 1 (µg/g)	Mean Concentration (µg/g)	Recovery %	CV%
Bile	10	8.965	9.285	9.125	91%	2%
Duodenum	10	9.244	8.896	9.070	91%	3%
Jejunum	10	9.379	7.423	8.401	84%	16%
Ileon	10	8.472	8.012	8.242	82%	4%
Caecum	10	8.331	9.108	8.720	87%	6%
Colon	10	8.082	9.334	8.708	87%	10%
Faeces	10	8.011	8.325	8.168	82%	3%

The LOD was estimated from the apparent signal of minocycline in blank chromatograms of each matrix (Figure 2). The five multiplied the apparent minocycline/IS ratio for each matrix was compared to the ratio of aqueous concentrations of the calibration curve, the LOD was set at 0.25 µg/g.

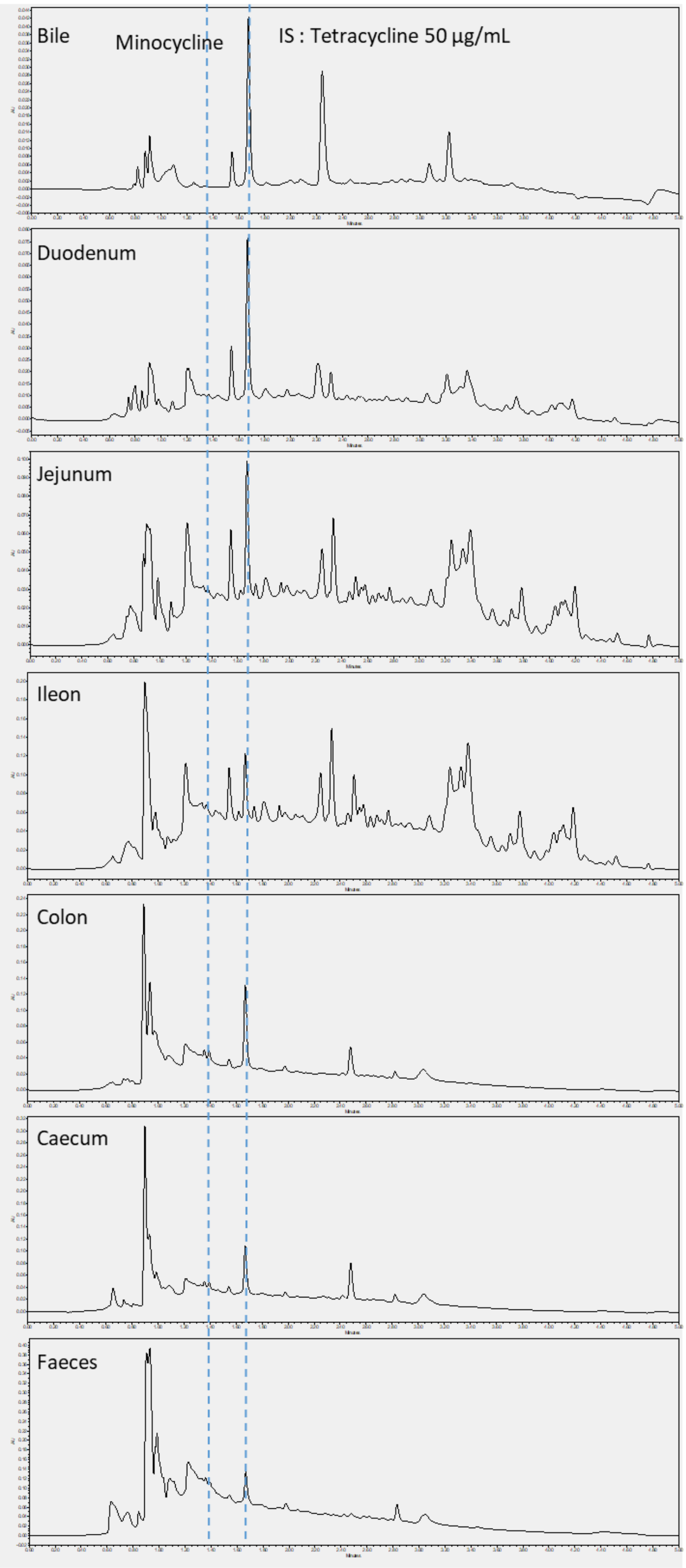

Figure 2: Chromatograms of extracted blank intestinal contents of pig spiked with internal standard (IS: 50 µL of tetracycline at 50 µg/mL).

References

EMEA/CHMP/EWP (2011). Guideline on bioanalytical method validation.
